# To Intervene or Observe: Accidental Ingestion of a Wireless Earbud in an Adult

**DOI:** 10.7759/cureus.102994

**Published:** 2026-02-04

**Authors:** Tanya Machado, Adarsh Nath, Joswin Madtha, Aafreen Vaz

**Affiliations:** 1 Emergency Medicine, SPARSH Hospital, Bengaluru, IND

**Keywords:** adult ingestion, foreign body, lithium battery, rare case report, wireless earbud

## Abstract

Foreign body ingestion in adults, particularly an electronic device containing a lithium-based battery, remains a challenging emergency department (ED) presentation, due to limited literature and specific guidelines. Management decisions depend on object characteristics, anatomical location, and symptomatology. We report the unusual case of an alert, 22-year-old female who accidentally ingested a single wireless earbud. On presentation to the ED within an hour of ingestion, she remained asymptomatic with stable vital signs throughout evaluation. An initial erect abdominal radiograph localized the foreign body to the second part of the duodenum. Following multidisciplinary discussion, a conservative “wait-and-watch” approach was adopted. Serial abdominal radiographs demonstrated gradual distal migration, confirming spontaneous passage of the device on day five. This case highlights the importance of individualized risk assessment and underscores the role of conservative management with close radiologic monitoring in selected asymptomatic patients, even when the ingested object is an electronic device containing a sealed lithium-ion battery.

## Introduction

Foreign body ingestion occurs predominantly in children [1], with adults comprising a minority of reported cases. Epidemiological data from the United States suggest a rising incidence of foreign body ingestion among adults, increasing from 3 to 5.3 per 100,000 persons between the years 2000 and 2017 [2]. Despite this observed trend, population-level data remain limited, and case reports describing ingestion of modern consumer electronic devices are exceedingly rare in the adult literature.

Management of foreign body ingestion in adults is influenced by both patient and object-related risk factors. Sharp or pointed objects, long or wide objects, multiple magnets, and batteries are associated with an increased risk of mucosal injury, obstruction, perforation, fistula formation, and bleeding [3]. Approximately 80-90% of ingested foreign bodies pass spontaneously, 10-20% require endoscopic removal [4], and fewer than 1% require surgical intervention [3]. A systematic review reported that despite 68% of patients having a foreign body impacted in the esophagus, a limited 4.5% experienced complications [5]. Current guidelines recommend urgent endoscopic intervention for esophageal foreign bodies or high-risk objects, while conservative observation may be appropriate once a blunt object has passed beyond the pylorus in an asymptomatic patient [6].

To the best of our knowledge, this case represents the first reported case of accidental ingestion of a wireless earbud in an adult. Although specific earbud models may minutely vary in shape, most wireless earbuds usually contain a sealed lithium-ion polymer battery within a rigid plastic case that has a smooth contour. Given the absence of device-specific guidance for the ingestion of wireless earbuds, this case provides pragmatic insight into clinical decision-making and suggests that existing foreign-body ingestion guidelines may be cautiously extrapolated to select electronic devices when structural integrity is preserved and the patient remains clinically stable.

## Case presentation

A 22-year-old female presented to the emergency department (ED) approximately one hour after accidentally ingesting a single wireless earbud at around 2:00 AM. The ingestion occurred in a dimly lit environment when she mistook the device for an oral analgesic tablet that she intended to take for transient abdominal discomfort experienced earlier at home.

On presentation to the emergency department, the patient was alert, oriented, hemodynamically stable, and asymptomatic. She denied dysphagia, odynophagia, chest pain, dyspnea, nausea, vomiting, abdominal pain, hematemesis, or melena at the time of evaluation. Physical examination revealed a soft, non-distended, and non-tender abdomen without guarding or rigidity. Cardiovascular and respiratory examinations were unremarkable. In view of her clinical stability and absence of systemic or toxic features, no laboratory investigations were obtained.

**Figure 1:**
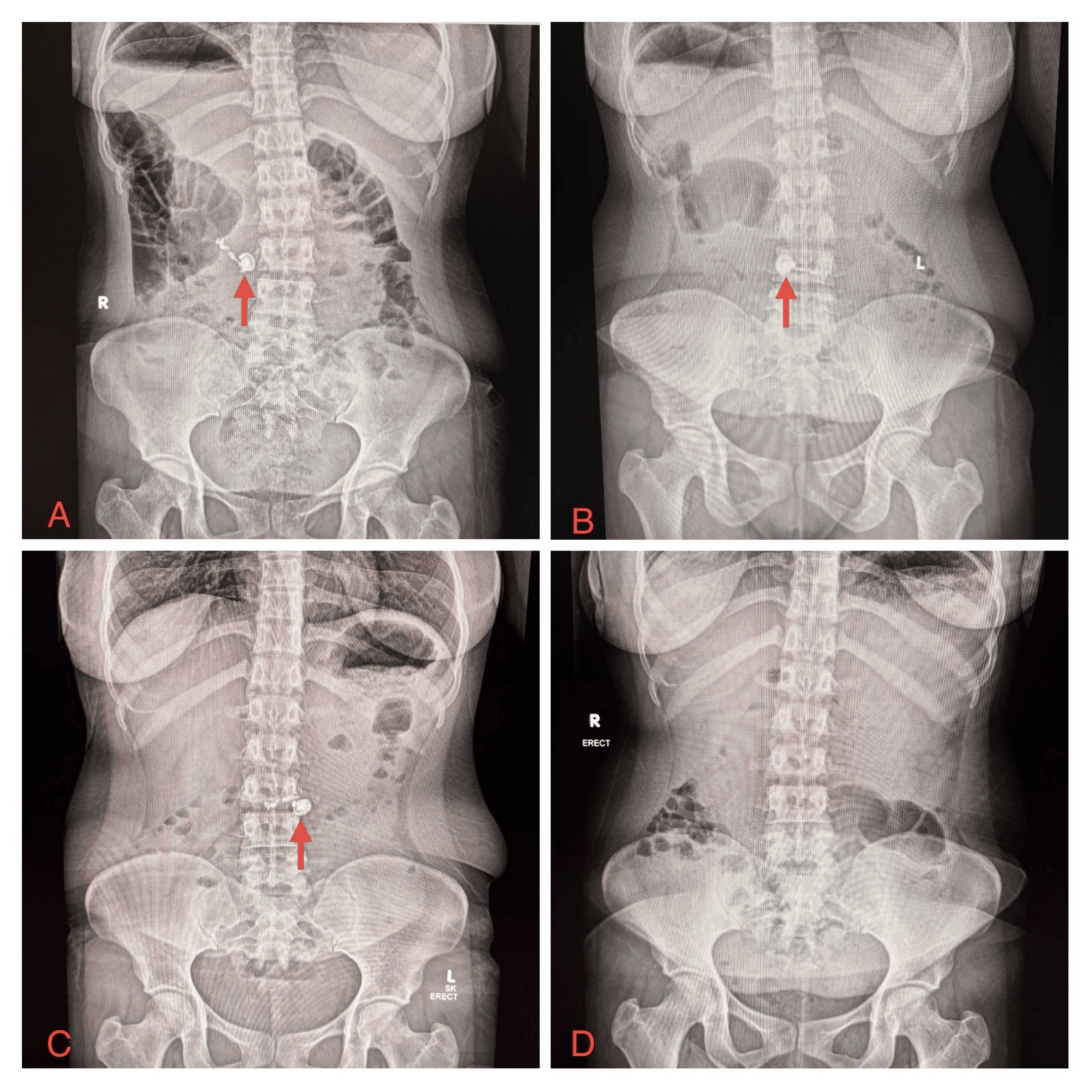
Serial erect abdominal radiographs demonstrating the progression and spontaneous passage of an ingested wireless earbud (A) Erect abdominal radiograph obtained at presentation demonstrating a single radiopaque foreign body consistent with a wireless earbud projected over the second part of the duodenum (red arrow). (B) Repeat erect abdominal radiograph at six hours post-ingestion demonstrating distal migration of foreign body to the region of the duodenojejunal junction (red arrow). (C) Follow-up erect abdominal radiograph at twelve hours post-ingestion demonstrating transient stagnation of the foreign body within the proximal jejunum (red arrow). (D) Follow-up erect abdominal radiograph on day five post-ingestion demonstrating complete passage of the foreign body, with no residual radiopaque density. R, right; L, left; SK, skull-knee direction of the X-ray beam.

Given the ingestion of a battery-containing electronic device, an erect abdominal radiograph was obtained to localize the foreign body. Imaging demonstrated a single radiopaque foreign body in the second part of the duodenum, as shown in Figure 1A.

Prompt consultation with a medical gastroenterologist was obtained. Following multidisciplinary discussion, a conservative management strategy with close clinical observation and serial radiographic monitoring was favored over urgent endoscopic retrieval. Factors supporting this “wait-and-watch” approach included the object having already progressed beyond the pylorus, thereby reducing the risk of prolonged gastric exposure and battery-related injury. Additional factors supporting a conservative approach included the foreign bodies’ blunt contour on imaging and the patient’s persistent asymptomatic status.

A repeat erect abdominal radiograph obtained six hours later demonstrated distal migration of the foreign body to the duodenojejunal junction (Figure 1B). Given her continued clinical stability and absence of concerning features, the patient was discharged from the emergency department in accordance with established management guidelines for foreign body ingestion. She was given clear instructions to return in the event of abdominal pain, vomiting, hematemesis, or melena, and advised outpatient follow-up with medical gastroenterology.

At twelve hours post-ingestion, the patient presented for outpatient review. Repeat imaging revealed transient stagnation of the device in the proximal jejunum (Figure 1C). Multidisciplinary assessment confirmed the device to be radiologically intact, with no evidence of obstruction or perforation, and expectant conservative management was continued. A planned follow-up abdominal radiograph performed on day five post-ingestion demonstrated no visible foreign body, confirming spontaneous passage of the device (Figure 1D). The patient remained asymptomatic throughout the course, and no endoscopic or surgical intervention was required.

## Discussion

In adults, ingestion is most commonly associated with psychiatric illness, alcohol or substance intoxication, incarceration, and, less frequently, accidental ingestion [4]. Commonly ingested foreign bodies include fish bones (29.3%) and food boluses (27.5%), with the esophagus being the predominant site of impaction (68.1%) [5]. 

Potential complications of foreign body ingestion include mucosal ulceration, obstruction, perforation, hemorrhage, and infection. Battery-containing objects raise additional concerns due to the risk of chemical injury from alkaline leakage, thermal injury, and electrical discharge [6]. Studies among children have shown that button batteries can cause severe tissue necrosis within hours, particularly when impacted in the esophagus [7,8]. The likelihood of causing morbidity or mortality depends on the characteristics of the foreign body [9]. However, the risk profile changes significantly once a battery or battery-containing device passes beyond the stomach [8,10]. Multiple studies indicate that batteries located distal to the pylorus in asymptomatic patients can often be managed conservatively with close observation [7,11]. Additionally, these risks were of limited concern in the management and prognosis of our patient, given that she had ingested a lithium-ion polymer battery.

Wireless Bluetooth earbuds contain a miniature lithium-ion polymer rechargeable battery, which is structurally distinct from the button cell batteries commonly implicated in pediatric ingestions. Button cell batteries cause injury primarily through electrochemical hydrolysis, resulting in rapid alkaline injury to the esophageal mucosa. In contrast, these lithium-ion polymer batteries are further encapsulated within sealed plastic or metal casings, designed to prevent leakage or electric discharge [12]. When ingested intact, the risk of chemical caustic injury is therefore substantially lower, with primary clinical concerns instead related to mechanical obstruction, pressure necrosis, perforation, or aspiration.

In concordance with the European Society of Gastrointestinal Endoscopy (ESGE) guidelines, plain radiography was chosen as the first-line imaging modality, given the non-bony nature of the ingested foreign body [6]. Plain radiography remains the first-line imaging modality for radiopaque foreign bodies and is recommended for initial localization and serial monitoring [13]. Computed tomography is recommended only in cases of suspected perforation that may require surgical management. Foreign body presentations to the ED within 24 hours of ingestion that are sharp-pointed objects, magnets, batteries, and large objects require therapeutic esophagogastroduodenoscopy [6]. ESGE guidelines in support of the “wait-and-watch” approach with serial imaging when the object is distal, non-sharp, and not associated with clinical deterioration [6] guided the management of this patient.

Endoscopic and surgical interventions carry inherent risks, including perforation, bleeding, and anesthesia-related complications [14]. Conversely, prolonged retention of a foreign body carries a minor yet serious risk of delayed obstruction or mucosal injury [15]. Long-term follow-up studies suggest that patients managed conservatively, when appropriately selected, experience excellent outcomes with minimal sequelae [16]. In this case, conservative management was indicated, given the distal progression of the foreign body and the lithium-ion polymer battery with intact casing. It avoided unnecessary invasive procedures and associated risks while achieving complete resolution without complications, supporting the safety of observation in selected adult patients.

## Conclusions

This case demonstrates that accidental ingestion of a wireless earbud in an adult may be safely managed with a conservative, multidisciplinary approach when the patient is asymptomatic and the object has progressed beyond the pylorus. Despite theoretical concerns regarding battery-related chemical injury, careful adherence to established foreign body ingestion guidelines, combined with serial radiographic surveillance and close clinical monitoring, facilitated spontaneous passage without adverse outcomes. This report underscores the importance of individualized risk assessment in guiding management decisions for such novel ingestion scenarios in adults.
